# Mr. Madden's Travels. Plague, &c.

**Published:** 1829-10-01

**Authors:** 


### TRAVELS IN TURKEY, EGYPT, NUBIA, AND PALESTINE. By *R. R. Madden*, Member of the Royal College of Surgeons.

[ART. II.]

IN our last Number we did little more than introduce Mr. Madden to the notice of our readers, and left him in the "city of the plague," Alexandria, where he resided two years. He arrived in that city just at the period when the pestilence was commencing its ravages.

"The plague daily increased in violence, eighteen a day of the natives perished, and few days passed over without the death of Europeans. For so small a population as that of Alexandria, say sixteen thousand souls, the mortality was considerable: every house was shut up, the servants were not suffered to go out, money was passed through vinegar before it was touched, letters were smoked, papers were handled with tongues, passengers in the streets poked unwary strangers with their sticks, to avoid communication, people thronged round the doctors' shops, to know how many died in the night, the plague was discussed at breakfast, contagion was described at dinner, buboes and carbuncles (horresco referens) were our themes at supper. The laws of infection were handled by young ladies in the drawing room; 'a cat could communicate the plague, but a dog was less dangerous; an ass was a pestiferous animal, but a horse was non-contagious. Fresh bread was highly susceptible, but butchers' meat was non-productive.' If you looked at a man, he felt his groin; if you complained of a head-ach, there was a general flight; if you went abroad with a sallow cheek, the people fled in all directions; if you touched the skirt of a Christian's coat, you raised his choler, and if you talked of M'Clean, your intellect was suspected to be impaired. Heaven preserve you from a quarantine in Egypt! It is not the death of one's neighbours which is so overcoming; I am now accustomed to coffins; I can hear of a case next door without a sympathetic pain in my axilla; but it is the horror of eternally hearing of plague; it is the terror of contagion, which is depicted in every face; it is the presentation of pestilential apparitions and discourses to the eye and to the ear, morning, noon, and night, which make a house in quarantine a lazar domicile, for the anticipation of death and the anatomy of melancholy." 253.

Of all human horrors, earth has nothing to compare with the dismay depicted on the features of the sick in the pest-houses. Our author endeavoured to persuade the Doctor of one of them (M. Marpurgo) to employ brandy, wine, and bark, but he refused, alledging that the celebrated jew physician here had made use of much stronger stimulants, namely, *prussic*

*acid* and *nux vomica*, without success. There was no resisting this argument.

After remarking that, in every stage of plague, Nature appears to lie prostrate under the influence of a poisonous miasma, and ultimately sinks from want of force to draw out the eruptions on the surface, Mr. M. proceeds thus:—

"The bubo recedes, or the carbuncle diminishes, or neither appear at all externally; but they have seized on the internal vital organs, and the immediate cause of death has been shewn by dissection to have been carbuncles on the liver, lungs, spleen, or mesenteric glands; in short, it appears that the whole glandular system is the seat of the disease. I have seen all the different species of plague enumerated by Russel and the French authors, and I have no hesitation in pronouncing all these different species of plague to be the symptoms of *one class* only; and I assert there is but one indication to fulfil, namely, to assist nature to expel the poison by strengthening the exhausted powers of the constitution, and enabling it to throw out the morbific matter. By what means is this to be done,—whether by emetics, by purgatives, by bleeding, by calomel, by mercurial unction, or by oil friction? There is none of these means I have not tried, and out of the first eleven patients so treated I lost nine. I had recourse to another mode of cure: I certainly did not see it employed by any other person; and although the propriety of adopting it has been glanced at by Assaline, it never was carried into effect, or at least pushed to the extent at which I found it an invaluable plan of treatment. I do believe what I now inform you of will one day or other acquire me some little credit, and that I shall be considered, in some sort, instrumental to the conquest of a disease which hitherto has been deemed almost invincible.

"Strong stimulants diffusible and permanent, I now tried; I commenced with wine and brandy the first moment I saw the patient; whether the eye was suffused, the cheek flushed, and the skin arid, or the low delirium set in or not, I administered it in the following manner. The first dose was a tumbler of hot brandy and water, about one-third spirit, this sometimes was vomited, and again repeated; the second time it usually remained on the stomach, and in the course of two hours it generally produced perspiration, even after James's powder had failed: two or three hours after the first dose, another was exhibited, and the patient would feel less of the burning pain at the heart; if vomiting supervened, it was again repeated: and during the day, it was now given every four or six hours, according to circumstances. The buboes commonly increased in size, and profuse sweating was often followed by *petechiæ*, or livid spots on the chest; when I saw this, I was always sure of my patient. The second day I increased the strength of the dose; instead of one-third spirit, I gave one-half, every eight hours, no intoxication came on, but a lethargic drowsiness was common enough, continuing till the perspiration broke out, or carbuncles appeared externally. If on the third day the patient was decidedly better, I kept up the excitement with strong cyprus wine, in frequent but small doses of two tablespoonsful every two hours; but if the bad symptoms were unabated, I continued to give the hot brandy and water, in increased quantities, till some decided change took place: this active treatment it was seldom necessary to pursue beyond the sixth day. Indeed, in plague, if the patient live to the sixth day, he is very likely to recover; but the third day is that which is most to be feared. The only other treatment was once or twice opening the bowels with enemas, for purgatives by the mouth do no service; and spunging the body frequently with vinegar and water; the head was constantly kept soaked with towels dipped in vinegar, and the buboes were poulticed with very hot cataplasms, sufficiently hot to give pain, and they were allowed to burst spontaneously." 269.

Under the above treatment, 75 per cent. recovered. In Candia, out of nine cases which our author attended, five recovered, and some of these were nearly hopeless when he first saw them. When he arrived in Alexandria, he proposed to Mr. Salt and to Mr. Thurburn to attend plague patients exclusively for one season, with an hospital of his own; but this could not be effected. Mr. M. then contented himself with daily attendance at the Plague Hospital—not under the idea of the disease being non-contagious—far from it; but because he did not think it, under ordinary circumstances, so contagious as people imagined.

"Plague, under ordinary circumstances, is with difficulty communicated; I say so advisedly: but, like all other diseases, it may be rendered highly contagious, by crouding a number of patients into a small chamber, ill ventilated, filthy, and offensive. I believe that there are very few disorders which may not become contagious under such circumstances. In Rome, I have seen pulmonary consumption rendered infectious by the closeness of the sick chamber (for the air is always carefully excluded in this disease), and the *damp heat* of the climate. The Romans all know this disease to be highly infectious; yet the assertion will appear monstrous in England. I have known repeated instances of persons going into a plague chamber, where the doors and windows have been kept closed, and the foulness of the atmosphere has been perceptible to the sense, exclaiming that their head was bursting with sudden pain, and they have gone home, and went abroad no more.

"I do not say that contagion is generated *only* in the close, ill ventilated chamber; but I say it is there augmented, in violence and virulence. Perhaps it may be inferred, that plague is therefore only communicated by the breath, and not by contact. I have reason to know it is communicated by both, in the instance of my own servant; he gave the disease to his brother by shaking hands, and the brother communicated it, through the atmosphere, to a lodger who casually entered the apartment for a moment, yet touched no person in it.

"In a close chamber, the woollen clothes (above all) become saturated with the contaminated air; and if persons who waited on the sick, entered without any other clothing than an oil-skin garment, sprinkled well with vinegar, the danger of infection would be diminished one half.

"In a word, plague under all circumstances is contagious, but under some, far more so than under others. In a well ventilated chamber, where the bed-clothes are shifted daily, where the floor is washed daily, *and a fire kept constantly in the apartment (this I consider the most important agent of all in carrying off the foul air)* there is hardly any peril in approaching the bedside of the sick, avoiding his breath, and suffering no part of one's dress to touch the bed-clothes; but four feet from the bed of the plague patient, in an airy room, there is no danger whatever. The miasma, I have ascertained, by much observation, (so far as an invisible agent is amenable to observation or experience) does not extend beyond a very few feet from its source; I would say, not four feet from the bedside, and then it becomes so diluted by the surrounding atmosphere as to prove innoxious." 272.

Mr. M. never entered the Plague Hospital without having the doors and windows thrown open ten minutes previously. He also took two glasses of Cyprus wine, and while in the hospital continued to smoke a cigar or pipe. He strenuously cautions the physician against unnecessarily exposing himself to the contagion, by stooping over the sick, inhaling the patient's breath, and feeling the tumours and the pulse. Nothing is to be gained from these investigations.

Whether it be a casual coincidence or not that the commencement and termination of the plague correspond with the rise and fall of the Nile, Mr. M. does not pretend to determine—but such is the fact. The plague commences in March, and declines at the Summer solstice—ceasing when the inundation is established—and beginning again when the lands have been drained.

"If I were to suffer myself to hazard a conjecture, it would be that the miasma, which produces plague, is not generated in those parts which are inundated by the Nile; that neither the inundation nor the draining off of the waters has any thing to do with the generation of the miasma; but that the peculiar condition of the atmosphere, which is produced by the changes of the Nile on the surface of the country, has a material influence on the development and dissipation of the miasma: in short, that the atmosphere is only the *medium* of the poisonous miasma which has its *origin* elsewhere.

"Both plague and malaria have their origin in putrefaction, exhaling an invisible vapour, which can only be estimated by its consequences. Malaria originates in the decomposition of vegetable matter. *Plague miasma, according to my opinion, originates in the putrefaction of animal matter.* The production of both, of course, depends on certain states of moisture and heat, which in other places of even a damper climate and a higher temperature, are wanting to the generation of these diseases." 284.

It is curious that the Jewish quarter in Rome is the last assailed by malaria, while the Jewish quarters in the Levant are always the first affected by plague. In Turkish towns the filth is generally beyond conception—and, in our author's opinion, it is the pre-eminent accumulation of these immundicities in Egypt and Turkey, aided by a certain disposition of the atmosphere, which confines the plague to these countries.

"In all Turkish towns, as well as those of Egypt, the narrow streets are never cleansed; there are no sewers, and the butchers kill their meat in the public streets: if the blood flow beyond the threshold of their door, they sweep it to their neighbours'. Dead dogs, cats, and rats are constantly putrefying in the middle of the streets; and in the narrow lanes of Alexandria, I have seen the carrion of camels and asses lying in the thoroughfare, and contaminating the air of all the neighbourhood.

"If these causes, in the purlieus of St. Giles's and Covent Garden would be esteemed sufficient to generate the poison of typhus fever, and this disease is always to be found in summer in these places, how much more should the miasma, arising from animal putrefaction, be thought likely to produce the worst stage of typhus—the plague, in the burning streets of Cairo and Alexandria?" 286.

Our author is convinced that, however the miasma may be first produced, the disease which follows its introduction into the body, namely, the plague, "is slightly contagious under ordinary circumstances; but that, from want of ventilation and cleanliness, it becomes highly infectious"—that it never can exist for any length of time in a country where cleanliness prevails and a good police exists—that an over-rigorous quarantine, causing people to be confined to their houses, is the most likely way to continue the pestilence—but that wholesome sanitary laws, above all directed to the cleansing of sewers, &c. are highly beneficial, and ought never to be dispensed with—finally, that a stimulant mode of treatment should be adopted, when the disease occurs, and depletive measures avoided.

In a subsequent letter, our enterprising traveller has touched on the principal diseases of the East, which, besides the plague, are chiefly dysentery, ophthalmia, bilious remittent fever, ague, and acute inflammatory fever. On these he has made some excellent observations, and he has laid down a code of hygiene, for the guidance of oriental travellers which they would do well to study. It is melancholy to contemplate the list of victims to an Eastern climate which a few years have presented. "Few weeks pass over without swelling the history of ill-starred enterprise with some new deaths to supersede the less recent obituary, in which are found the names of Bowden, Burckhard, and Belzoni." In lower Egypt Mr. Madden has seen three English medical men in two years "carried off by their own injudicious medication." Our traveller is a great advocate for temperance, nay, even abstemiousness, while undergoing fatigue in hot climates. The following passage is worthy of notice.

"When the traveller is actually *en route*, he should be more abstemious than usual; and whatever he eats should be of the plainest kind; when he halts for the night, he should repose a couple of hours before his meal, and it is only after sun-set he should indulge in his libations, albeit they consist in spring water. He will observe that the Arabs in the Desert seldom or never drink during the day, and imitate in some sort the economy of their camels, in laying in a stock of water over night. When exposed to the burning sun of the Desert, the more a man drinks the more frequent his desire to allay his thirst; and the more peril he encounters from obstructing insensible perspiration.

"It is almost incredible on what a small quantity of food the Bedouins subsist. I found the ordinary allowance of a Bisharein Arab did not exceed twelve ounces a day of black bread and salt cheese, with a few dried dates; and there was hardly any disease amongst them. I never saw hardier people; their frames were slender, but their activity was surprising, and their fine black eyes sparkled with intelligence and animation. Had they lived in towns, they would have consumed four times as much food, and their infirmities would have been at least fourfold." 386.

Dysentery is a great scourge of European travellers in the East. Mr. Madden opened the bodies of seven English sailors who died of dysentery in Alexandria, and in almost every case there was disease of the liver, actual or incipient. The two principal modes of treatment are, copious and repeated bleeding—or mercurialisation. Mr. Madden had reason to prefer the latter.

"If any drug deserve the name of specific, calomel, in dysentery, has a title to it. I seldom gave less than twelve grains, thrice a day, and very often twenty. An effect once produced on the salivary glands, the most urgent symptoms cease as if by magic." 392.

Ophthalmia was only to be subdued by large bleedings from the arm, repeated till every symptom of inflammation subsided.

The bilious remittent fever is the most formidable disease of all, and many are the European victims to its merciless virulence.

"Wherever vegetable and animal decomposition are going on, wherever date trees are thickly clustered, wherever there is a saline incrustation on the soil, and a rank verdure in the vicinity, there that miasma is to be apprehended which produces bilious remittent fever. The distinguishing symptoms are, irritability of the stomach, oppression about that region, early delirium, and a lurid yellowness of the eyes; the subsequent symptoms are, vomiting of dark and viscid bile, fulness about the left side, intense headach, small rapid pulse, and often diarrhœa." 393.

The treatment, Mr. M. observes, must be active, for the fever runs its course in from three to seven days—rarely stretching out to twenty.

"The first measure should be blood-letting from fifteen to twenty ounces, repeated if necessary next day, and the following ten grains of calomel and one of opium three times a day, for four or five days, or till salivation is produced: every night six grains of James's powder, no emetics, but daily doses of castor oil or colocynth are necessary. When the fever runs very high and the skin is parched and burning, the body should be sponged with diluted vinegar. This treatment I have seldom seen fail, but where the inflammation of the irritable stomach, and the gorged liver is aggravated by bark, given in ignorance of the disease, death has generally ensued." 394.

But our limits are exceeded, and we must here take leave of our most enterprising, amusing, and intelligent traveller. We believe that very few will open these volumes without perusing them to the end, and that without *skipping* a single page. These "personal narratives," under the influence of Mr. Madden's pen, combine the interest excited by the romantic adventures of a Crusoe, with the deep researches of a Humbolt—divested of fabulous feeling on one hand, and *fatiguing science* on the other.

Mr. Madden has had an ample range of adventures, and has seen enough of foreign countries. We trust that our talented and courageous HACKKIM will be in as great request in Bolton Row, as he was in the Harems of Stamboul, the pest-houses of Alexandria, the tents of the Bedouins, or the camps of the Pachas.

